# A phase 1 safety and feasibility trial of a ketogenic diet plus standard of care for patients with recently diagnosed glioblastoma

**DOI:** 10.1038/s41598-025-06675-6

**Published:** 2025-07-01

**Authors:** L. J. Amaral, Gillian Gresham, Sungjin Kim, Mourad Tighiouart, Thomas A. Nelson, Amelia Welborn, Laura Lockshon, Brandon Noorvash, Jeremy D. Rudnick, Scott A. Irwin, Stephen J. Freedland, Jethro Hu

**Affiliations:** 1 Cedars-Sinai Cancer, Cedars-Sinai Medical Center, Los Angeles, CA USA; 2 Department of Medicine, Cedars-Sinai Medical Center, Los Angeles, CA USA; 3 Department of Neuro-Oncology, Massachusetts General Hospital, Harvard Medical School, Boston, MA USA; 4 Biostatistics and Bioinformatics Research Center, Cedars-Sinai Medical Center, Los Angeles, CA USA; 5 Department of Health, University of California Los Angeles, Los Angeles, CA USA; 6 Center for Digestive Health, Virginia Mason Medical Center, Seattle, WA USA; 7 Cancer Research Center Health Equity, Cedars-Sinai Medical Center, Los Angeles, CA USA; 8 Department of Medicine, Neurology and Neurosurgery, Cedars-Sinai Medical Center, Los Angeles, CA USA; 9 Department of Psychiatry and Behavioral Neurosciences, Cedars-Sinai Medical Center, Los Angeles, CA USA; 10 Center for Integrated Research in Cancer and Lifestyle, Cedars-Sinai Medical Center, Los Angeles, CA USA; 11 Section of Urology, Durham VA Medical Center, Durham, NC USA; 12 Cedars-Sinai Cancer Patient and Family Support Program, Cedars-Sinai Medical Center, Los Angeles, CA USA; 13 Department of Surgery, Cedars-Sinai Medical Center, Los Angeles, CA USA; 14 Departments of Medicine, Neurology, and Neurosurgery, Cedars-Sinai Cancer, Cedars Sinai Medical Center, 8700 Beverly Blvd, Los Angeles, CA 90048 USA

**Keywords:** Glioblastoma, Ketogenic diets, Ketosis, Warburg effect, Cancer metabolism, Supportive care, Physical activity, Quality of life, Cancer, Cancer metabolism, CNS cancer, Oncology

## Abstract

Despite great interest, there is limited clinical evidence to support the use of a ketogenic diet (KD) for cancer patients. We conducted a single-arm phase 1 trial of a KD among patients with recently diagnosed glioblastoma (GBM) receiving standard-of-care (SOC) treatment. Adults with GBM within 3 months of diagnosis followed a supervised 16-week intervention of a 3:1 KD (Fat(g): Carbohydrate + Protein(g)) plus SOC chemoradiation. The primary outcome was safety, evaluated by weekly assessments of weight and body mass index (BMI). Secondary outcomes included feasibility (pre-specified as > 50% of patients maintaining blood ketone levels > 0.3 mM over 50% of study days), progression-free survival (PFS), overall survival (OS), health-related quality-of-life, and cognitive function. Twice daily blood glucose and ketones, weight/BMI, physical activity, and sleep were assessed by remote monitoring. Seventeen patients were evaluable: 53% women, median age 55, median Karnofsky Performance Status 85. All subjects met the primary safety objective with no instances of excessive weight loss or related serious adverse events. Adherence was high: all 17 patients maintained nutritional ketosis (≥ 0.3 mM/dL) > 50% of study days. Median PFS and OS were 12.9 months and 29.4 months from KD initiation respectively. Quality of Life, symptom control, and cognitive function remained stable or improved, although these did not reach statistical significance. This phase 1 trial demonstrates that KD is safe and feasible for GBM patients receiving SOC, may improve outcomes, and provides a foundation for an NCI-funded multicenter randomized diet trial to assess efficacy that is currently underway.

**Supplementary Information:**

The online version contains supplementary material available at 10.1038/s41598-025-06675-6.

## Introduction

Median survival for glioblastoma (GBM) is approximately 15–20 months and has improved only incrementally over the past 50 years^1^. In the face of such a dismal prognosis, it is not uncommon for patients to inquire about the role of health-related behaviors that fall within their sphere of control, particularly diet and exercise. There has been longstanding interest among the brain tumor community in the potential anti-cancer effects of a high fat / low carbohydrate, ketogenic diet (KD). Initially developed several decades ago as a treatment for epilepsy^2^, where its benefit is proven and its use continues to this day, interest in the anti-cancer effects of a low-carbohydrate diet dates back to the 1920s, when Otto Warburg noted that cancer cells exhibit increased glycolysis even under aerobic conditions^3^. More recent research has demonstrated that many cancers, including GBM, depend on glycolysis for the biosynthetic, bioenergetic, and signaling needs of oncogenesis^4^.

Given the confluence of clinical urgency, scientific rationale, lackluster conventional treatment, and the perception of a low barrier to entry (“it’s just a diet” and “everyone is doing it”), most neuro-oncologists have patients in their practice who have implemented KD in some form without dietitian support. And yet, beyond anecdotes, clinical evidence to support the use of KD for brain tumor patients is limited^5–8^.

In our own pilot project conducted in 2015, we evaluated KD in patients with CNS malignancies^9^. Twelve patients who had expressed interest in implementing KD as part of clinical care agreed to participate in a 120-day program that included close dietary supervision with regular monitoring of glucose and ketone levels, weight, and clinical outcomes. Diagnoses included GBM (*n* = 6), grade 2/3 astrocytoma (*n* = 5), and one patient with a grade 2 spinal cord astrocytoma. Ten of the 12 patients received concurrent treatment; two received supportive care only. As reported in our case series^9^all 12 patients were adherent to KD and were able to maintain average daily blood ketone levels greater than or equal to 0.5 mM^10^. Patients experienced a modest degree of weight loss (5–10% of Body Mass Index (BMI)). Notable improvements in energy, mood, neurocognitive function, and overall well-being were observed, as well as improved seizure control in some but not all patients. In 4 patients, imaging demonstrated a reduction in contrast enhancement or T2/FLAIR hyperintensity suggestive of radiographic response. Although these findings were observed in a heterogeneous group of patients, some of whom were on concurrent treatment, the positive experience compelled us to further investigate the potential role of KD for patients with recently diagnosed GBM in a phase 1 single-arm clinical trial. While a small number of early-phase trials have demonstrated that KD can be implemented safely for patients with other cancers, there remains a need for a prospective study that focuses on safety for brain tumor patients, as prospective data of KD for this specific patient population while going through cancer-directed treatment is still limited^11,12^. Additionally, given potential issues with diet adherence reported by other studies, assessment of feasibility was established as a key secondary objective in this study.

## Patients and methods

This trial was registered with ClinicalTrials.gov (NCT03451799) on 03/02/2018. The study was approved by the institutional review board at Cedars-Sinai, and patients were enrolled after providing written informed consent. Internal monitoring visits were performed by the Quality Management Core of the Cedars-Sinai Cancer Clinical Trials Office and reviewed by the Institutional Data and Safety Monitoring Committee. All experiments were performed in accordance with relevant guidelines and regulations.

### Study design

This single-institution, single-arm, phase 1 trial was conducted at Cedars-Sinai Medical Center according to International Conference for Harmonisation (ICH) guidelines and reported following CONSORT reporting guidelines for trials. As a phase 1 safety and feasibility trial, the study was not randomized. This was an open-label study; participants and study personnel were not blinded to the study intervention. Glucose/ketone meters and test strips were provided by Keto-Mojo (Napa, CA). Keto-Mojo was not involved in the conception, design, execution, or analysis of this study.

### Objectives

The primary objective of this phase 1 clinical trial was to assess the safety of a 16-week KD in patients with recently diagnosed glioblastoma receiving Standard of Care (SOC) treatment. The diet would be considered unsafe if, after 1 month on the diet, 20% of study participants experienced a 10% decrease in weight or BMI resulting in a BMI < 18.5. Adverse events were monitored and recorded per Common Terminology Criteria for Adverse Events (CTCAE) Version 5.0 guidelines. Assessment of feasibility was a major secondary objective, operationally defined as > 50% of patients being able to maintain blood ketone levels > 0.3 mM for over 50% of days on study, starting 2 weeks after KD initiation^13^. Other secondary objectives included assessments of progression-free survival (PFS) and overall survival (OS), health-related quality-of life (QOL), cognitive function, and physical activity.

### Inclusion and exclusion criteria

Patients > 18 years of age with newly or recently diagnosed GBM were eligible for this study. Patients were eligible to enroll at any point from the time of initial diagnosis until the initiation of post-radiation adjuvant chemotherapy (3–4 weeks after the completion of radiation), providing a window of approximately 3 months from the time of diagnosis for patients to enroll. Patients with Karnofsky Performance Status (KPS) < 70, BMI < 22 kg/m^2^, disorders of lipid metabolism, medical comorbidities that would limit a patient’s ability to complete the intervention, or on > 8 mg dexamethasone daily (or other steroid equivalent) were not eligible for this trial. Although patients were not allowed to participate in other therapeutic trials while enrolled in this study, off-label therapy at the discretion of the treating clinician was permitted. Patients were recruited primarily from the brain tumor program at Cedars-Sinai Medical Center and referred to the study by the treating clinician or dietitian.

### Intervention

All patients received standard-of-care treatment consisting of maximal safe surgical resection, followed by involved field radiation therapy with concurrent temozolomide chemotherapy (~ 6 weeks), followed by adjuvant cycles of temozolomide given Days 1–5 of a 28-day cycle beginning 3–4 weeks after the completion of radiation (Supplemental Fig. 1). MRI scans were performed according to standard practice, typically every 8 weeks, beginning with an MRI scan performed shortly prior to initiating treatment with adjuvant cycles of temozolomide post-radiation.

All patients were supervised by a KD-trained research dietitian, providing necessary support throughout the study period. Prior to KD implementation, the study dietitian performed an initial evaluation of each patient, assessing for factors that may affect adherence such as weight, nutritional status, performance status, psychosocial support, age, sex, weight trends, and BMI. Pre-intervention education included a detailed description of KD, individually tailored meal plans, along with education on topics such as interpreting food labels, developing shopping lists, eating at restaurants, and using glucose/ketone monitors. Patients were also provided guidance on managing common effects of KD, such as nausea, constipation, diarrhea, loss of appetite, and flu-like symptoms.

Patients were provided with glucose/ketone meters and test strips for home use and were instructed to check blood glucose and ketone levels twice daily. Over days 1–7 (depending on their baseline diet), patients were converted from their baseline diet to a supervised KD with a goal of a 3:1 ratio between grams of fat to grams of carbohydrates plus protein based on previous studies that utilized this ratio to achieve ketosis^14^. Patients were monitored by the dietitian at least weekly, either in-person or via telehealth. During each dietitian assessment, careful evaluations of the past week’s glucose/ketone measurements, patient symptoms, hydration status, and weight trends were performed, as it is typical for patients initiating KD to lose weight. Dietary plans were adjusted over time based on factors such as labs, appetite, symptoms, weight trends, and food preference.

### Assessments

Glucose and ketone monitoring data were used to assess adherence to the diet, total days in ketosis (as defined as $\:\ge\:$0.3mM), and calculate the Glucose-Ketone Index (GKI, defined as the ratio of glucose (mM) to ketone (mM) level) over the study period. Remotely monitored weight and daily activity (physical activity, sedentary time, and sleep) were assessed using a Bluetooth smart scale (Fitbit Aria; San Francisco, CA) and a wrist-worn wearable activity monitor (Fitbit Charge HR 3). Data were synced to patient devices using Bluetooth technology and combined with the remotely assessed glucose and ketone meters using HeadsUpHealth (Scottsdale, AZ). Patients were given the option to keep the glucose/ketone meters and Fitbit activity monitors at the end of the study as incentives to participate and adhere to study intervention.

Imaging responses were assessed using modified Response Assessment in Neuro-oncology (RANO) criteria^15^. PFS was defined as the time from initiation of the diet to disease progression (as defined by RANO) or death from any cause; OS was also calculated from time of KD initiation. The European Organisation for Research and Treatment of Cancer (EORTC) QLQ-C30 and QLQ BN-20 questionnaires were used to assess health-related QOL, and cognitive function was assessed by the Hopkins Verbal Learning Test (HVLT), Trail Making Test, Controlled Word Association Test (COWAT), and the Montreal Cognitive Assessment (MOCA). Fasting insulin and insulin like growth factor 1 (IGF-1) levels were evaluated every 4 weeks. Tumor molecular profiling was performed in accordance with routine clinical care at the sites where initial surgical intervention was performed. Tumor *IDH1* status was assessed by a combination of next generation sequencing and immunohistochemical analysis as part of standard-of-care. All study data were collected and managed in a HIPAA- and 21 CFR Part 11-compliant electronic Research Electronic Data Capture (REDCap) database.

### Power calculations

The primary objective was to evaluate the safety of the implementation of KD for 16 weeks where the diet would be considered unsafe if 20% of the study participants experienced a 10% decrease in their weight or body mass index resulting in BMI < 18.5 within 1 month from initiation of KD. Safety was evaluated by assessing weight change at each safety assessment (every 4 weeks) by the clinical team as well as weekly through Aria FitBit Bluetooth scale monitoring. Stopping rules for lack of safety were defined prior to trial initiation (See Appendix).

### Statistical analysis

Descriptive statistics were used to evaluate baseline patient characteristics, including sex as a biological variable, baseline age, race, performance status (KPS), and BMI. Individual outcomes (glucose, ketones, glucose/ketone index (GKI), weight, BMI) were modeled to examine changes over time using a generalized additive model for location, scale, and shape, (GAMLSS)^16^ with patient as a random effect and time using a penalized smoothing spline (p-spline), where the smoothing parameter was estimated using the local maximum likelihood method^17^. A Sinh-Arcsinh (SHASH) distribution and Box Cox-t distribution with an identity link function were used to model ketone and glucose levels, respectively. The goodness of fit of each model was examined using residuals such that the most adequate response distribution was chosen^18^. Estimated average values of ketone and glucose were graphically presented across time from baseline (day 0) to end of study (day 112). Changes in outcome measures over time using p-splines were assessed with the likelihood ratio test.

Kaplan-Meier estimates and log-rank tests were used to assess overall and progression-free survival distributions. Univariable Cox proportional hazards models were fit to evaluate the effect of baseline age, sex, extent of resection, performance status (KPS), baseline steroid use, *MGMT* promoter methylation status, glucose levels, and ketone levels on survival. For time-to-event analyses, time zero was defined as the time of diet initiation to date of death for overall survival, or date of first progression. All efforts were made to prevent missing data in the study. In the case that data were missing, an analytic plan for handling missing data was determined a priori and outlined in the study protocol. Statistical analyses were performed using Stata 15 (Texas) and R package version 4.0.5 with two-sided tests at a significance level of 0.05. Additional details on the statistical analysis plan are described in the appendix. The study protocol and statistical analysis plan are available in eSAP1.

## Results

### Study timeline

**Fig. 1:**
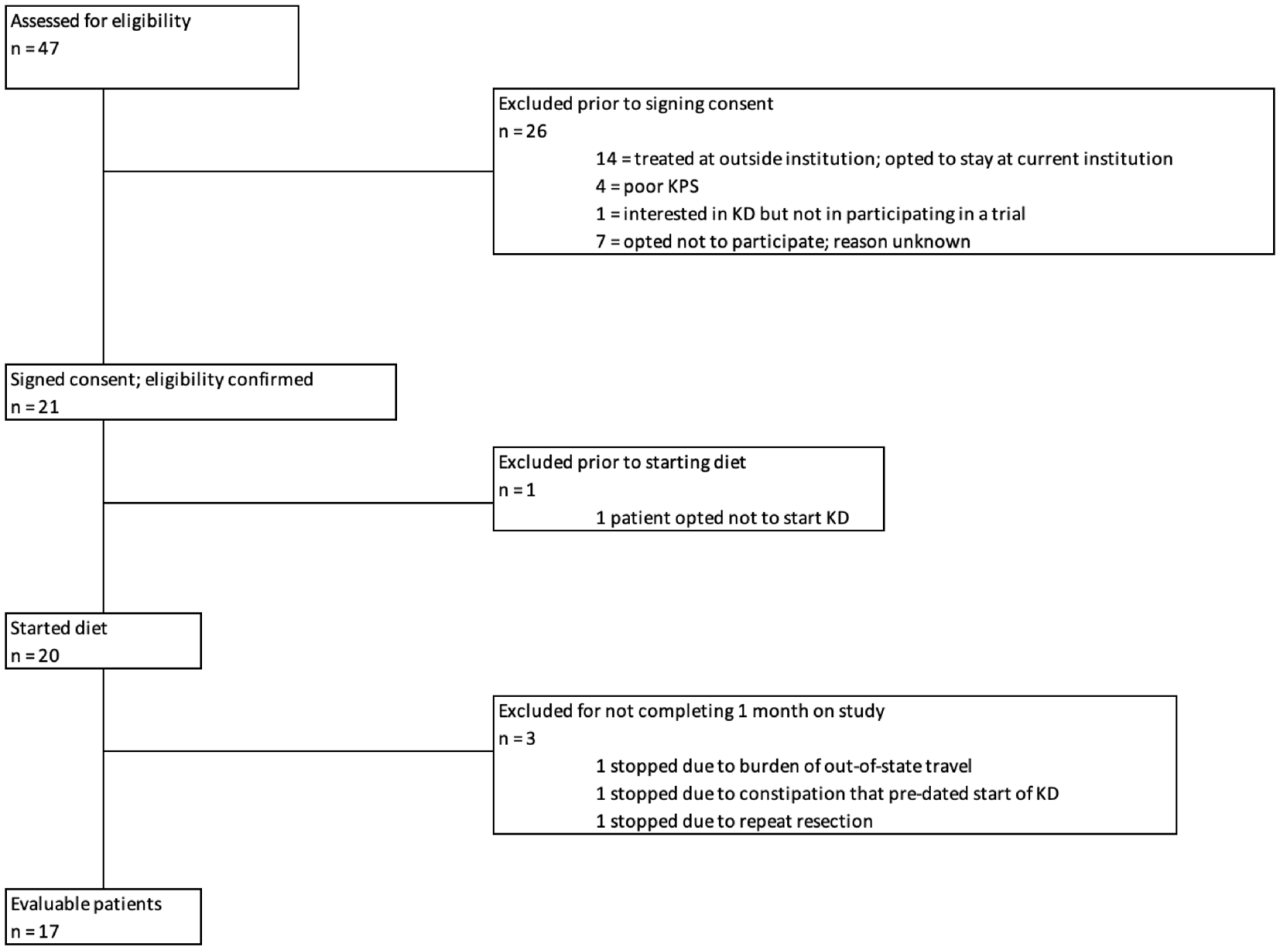
Modified CONSORT diagram.

From April 2018 until February 2021, 21 patients with recently diagnosed GBM consented to participate in the trial and 17 patients were eligible for analysis. In order to qualify for data analysis, patients had to have been on trial for 4 consistent weeks, therefore, four patients voluntarily withdrew before the 4 week threshold. (Fig. 1). Thirteen patients were diagnosed at our institution; four patients were diagnosed elsewhere and came to our institution to participate in this trial. From March 2020 through October 2020, trial accrual was impacted by the effect of the COVID-19 pandemic on clinical research operations at our institution.

### Patient characteristics

Patient characteristics are summarized in Table 1. Median age at enrollment for the 17 evaluable patients was 55 years old, with median KPS of 85. Sex was evenly distributed in this population with nine (53%) females and 8 males (47%) participating in the study. The majority (94%) of patients were non-Hispanic White. Nine patients underwent gross total resection prior to enrollment, 5 had subtotal resection, and 3 had biopsy only. Sixteen tumors were *IDH1* wild-type and 1 was *IDH1* mutant. The updated 2021 WHO Classification of Tumors of the Central Nervous System has re-categorized *IDH*-mutant, grade 4 astrocytoma as a separate entity from glioblastoma; at the time patients were enrolled on this trial, this was not the case^19^. Tumor *MGMT* promoter methylation was present in 7 tumors, absent in 9, and indeterminate for 1 patient.

**Table 1:** Baseline characteristics (*n* = 17).

Characteristic	*N* (%)
Sex	
Female	9 (53)
Male	8 (47)
Age Median (Range)	55 (26–71)
Race/Ethnicity	
Non-Hispanic White	16 (94)
Hispanic or Latinx	1 (6)
KPS Median (Range)	85 (70–90)
BMI Median (Range)	24.9 (21.5–32.4)
Prior surgery	
Complete resection	9 (53)
Partial resection	5 (29)
Biopsy only	3 (18)
Steroid use at baseline	
No	9 (52.3%)
Yes	8 (47.1%)
MGMT methylation status	
Unmethylated	9 (53)
Methylated	7 (41)
Indeterminate	1 (6)
IDH1 status	
Mutant	1 (6)
Wild-type	16 (94)

Twelve of the 17 evaluable patients completed the planned 16-week intervention. Five patients discontinued study participation early: 2 for disease progression, 1 due to grade 4 pneumonitis from off-label nivolumab, and 2 to participate in alternate therapeutic clinical trials despite absence of disease progression (though both patients continued KD off-trial).

### Safety, adherence, and tolerability

All 17 patients met the primary objective of safety as specified for this trial, with zero instances of excessive weight loss. Adherence to KD was high, with all patients (100%) maintaining nutritional ketosis (defined in this study as 0.3 mM) > 50% of study days. (Table 2.)

**Table 2:** Adherence and GKI data by individual patient with steroid use, over study period.

Individual patient	# days on study	% days in ketosis (*≥* 0.3 mM)	# days to enter ketosis	Average GKI*	% days GKI < 2	On Steroids^+^	Maximum dose of dexamethasone (mg)
A 1	111	100	1	1.9	56.8	N	N/A
B 4	56	99	1	4.8	7.1	N	N/A
C 5	112	99	1	4.1	4.5	N	N/A
D 6	106	98	1	5.2	1.9	N	N/A
E 7	85	98	1	6.3	1.2	Y	2
F 8	110	99	1	4.5	4.5	N	N/A
G 9	73	98	1	6.7	0	Y	10
H 10	104	52	2	17	0	Y	10
I 11	65	86	1	9	0	N	N/A
J 12	49	97	3	5.9	2	Y	0.5
K 13	112	97	1	4	13.4	N	N/A
L 14	114	88	1	8.9	10.5	Y	10
M 15	109	56	1	18.5	0	Y	4
N 17	112	53	2	17.4	1.7	Y	10
O 19	111	96	1	7.9	0.9	Y	2
P 20	112	100	1	7.5	0	N	N/A
Q 21	112	100	1	6.3	0.9	N	N/A

*GKI: ratio of glucose (mM/L) to ketone (mM/L) levels, where a GKI < 2 indicates high level of ketosis achieved.

^+^Steroid use indicative of use at any point in time during 16 week trial. N/A: Not Applicable

**Fig. 2:**
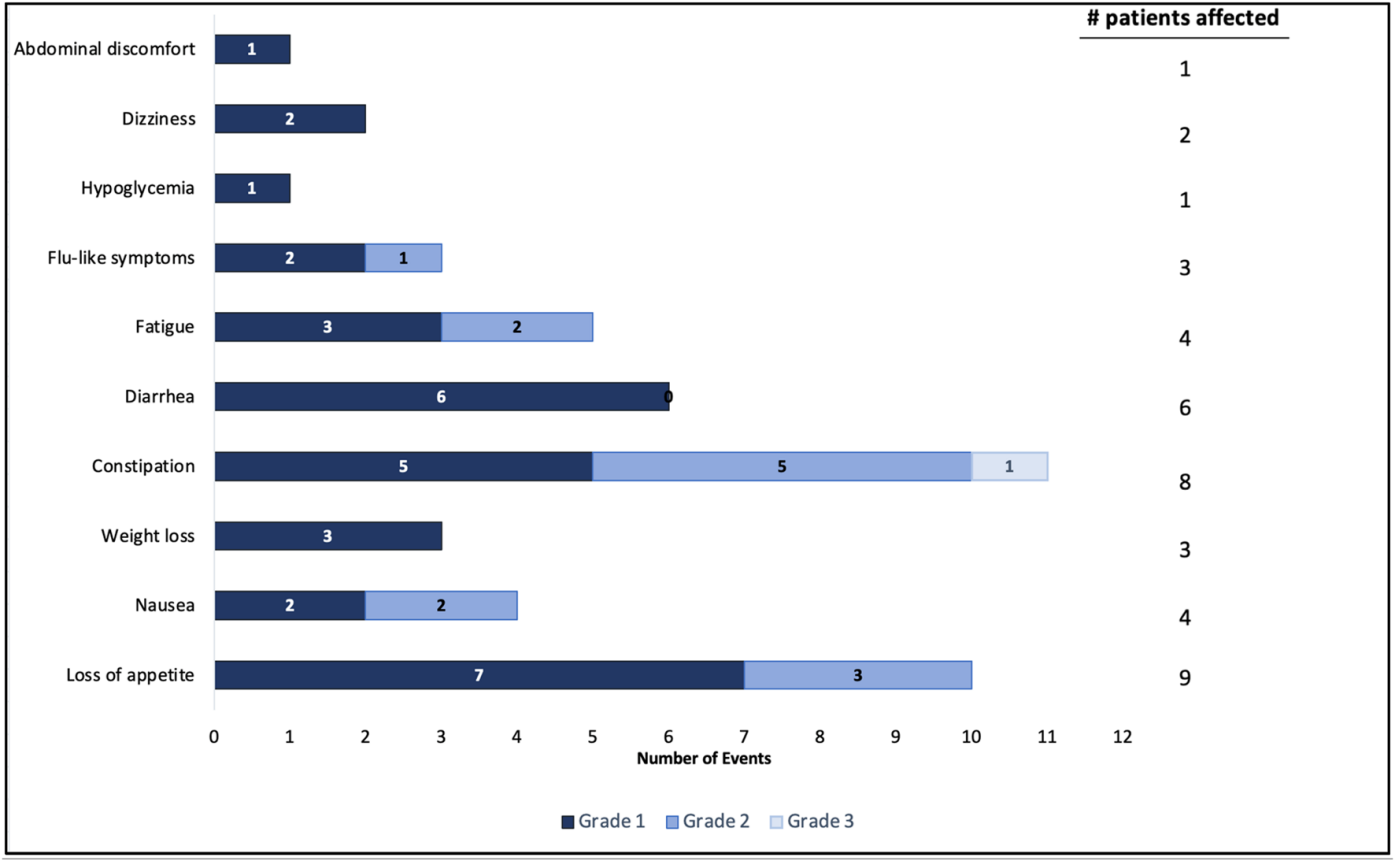
Adverse events possibly or probably attributable to KD*. *Evaluated in all patients who initiated diet (n=20); patients experienced more than one AE.

A total of 46 grade 1–3 clinician-rated Adverse Events (AEs) possibly or probably attributable to KD were experienced by 10 patients who initiated the diet; the remaining patients did not experience any diet-related AEs. (Fig. 2.) No patients came off study due to inability to tolerate the diet. The most common AE, loss of appetite, was observed in 9 patients, all grade 1 or 2 in severity. Grade 1 weight loss occurred in 3 patients. Five events of “flu-like symptoms” were observed, affecting 3 patients; one event was grade 2, the rest were grade 1 in severity. Eight patients experienced constipation while on trial (including 1 grade 3 event, the only AE > grade 2 possibly related to KD observed over the trial), 6 patients experienced diarrhea, and 4 patients experienced fatigue.

### Physiologic effects of KD

**Fig. 3:**
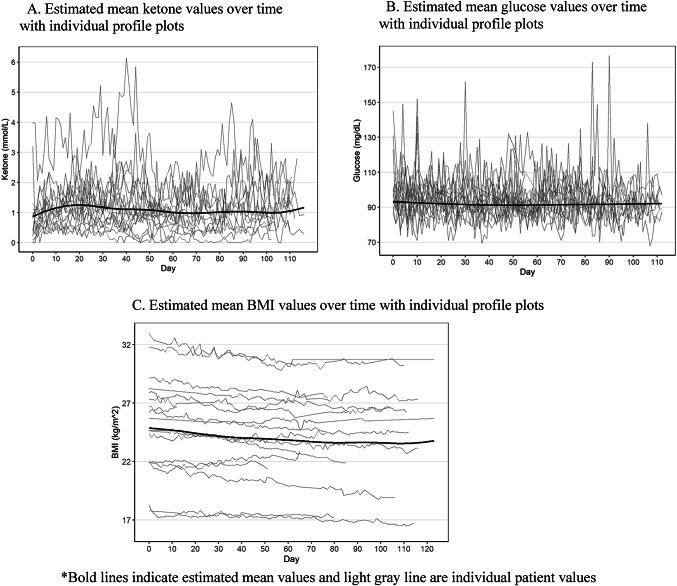
Physiologic effects of Keto diet on (**A**) Ketones, (**B**) Glucose, (**C**) BMI (*n* = 17).

A total of 3,126 ketone and glucose measures across 17 evaluable patients were obtained. The mean per-patient percentage of study days in ketosis was 97. On average, patients were able to achieve ketosis within 3 days of formal diet initiation, ranging from 1 to 3 days, although this metric is likely confounded by the eagerness of several patients to attempt to initiate KD “on their own” prior to their official baseline date. Figure 3 shows estimated mean ketone (Fig. 3A), glucose (Fig. 3B), and BMI values (Fig. 3C) with individual plots over the study duration. Changes in outcomes over time with 95% confidence intervals using p-splines are shown in Supplemental Fig. 2. There were statistically significant changes in ketone values (*p* < 0.001), glucose (*p* = 0.01), and GKI (*p* < 0.001). Statistically significant changes in remotely-monitored BMI and weight were also observed (*p* < 0.001); these changes did not meet the criteria for stopping diet. A decrease in serum IGF-1 levels over the 16-week period was observed from 401 ng/mL at baseline to 246 ng/mL at week 16 (*p* = 0.04). (Supplemental Fig. 3.) Fasting insulin levels decreased in the first 8 weeks, followed by an increase from weeks 12 to 16 (Supplemental Fig. 3). These findings are being explored further and will be reported separately.

There were 8 patients (47%) who were treated with steroids at any point over the course of the study (excluding topical steroids) and 4 patients who received > 8 mg dose of dexamethasone. Additionally, 1 patient was taking prednisone (40 mg) in addition to dexamethasone. Given the variability in steroid types and doses, we conducted an additional analysis comparing average GKI and % days in ketosis by receipt of any oral steroid as well as by maximum dose of dexamethasone received. Patients receiving any steroid had statistically significant lower % days in ketosis (mean: 79.8) compared to those not receiving steroids (97.6) (*p* = 0.03) and had higher average GKI values (11.1 vs. 5.3) (*p* = 0.01). Receipt of > 8 mg dexamethasone was also associated with statistically significant difference in average GKI values (12.5 vs. 6.6, *p* = 0.03) and % days in ketosis (94.2 vs. 72.7, *p* = 0.02).

### Survival

**Fig. 4:**
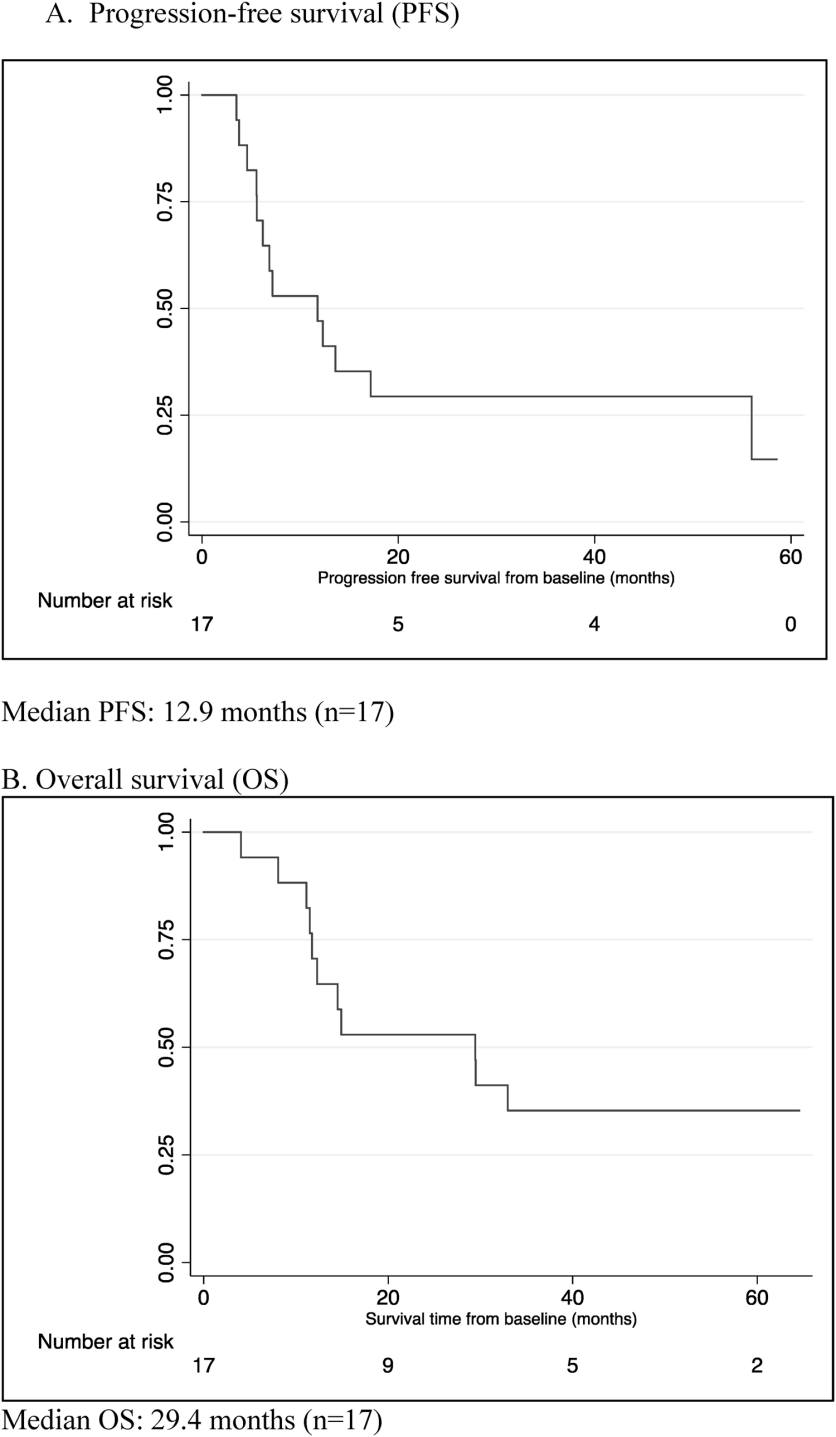
Kaplan-Meier estimate of progression-free survival and overall survival from KD initiation.

Calculated from the time of KD initiation (Day 1), median PFS for evaluable patients was 12.9 months and median OS was 29.4 months. Kaplan-Meier estimates for PFS and OS are shown in Fig. 4. When calculated from date of diagnosis, median PFS was 13.2 months and median OS was 30.5 months. In this small phase 1 trial, exploratory univariate survival analysis did not reveal statistically significant relationships between survival (either PFS or OS) and KPS at diagnosis, age, sex, statin use, extent of resection, or *MGMT* methylation status at baseline, and mean glucose levels, mean ketone levels, or mean GKI (Supplemental Table 1).

### Quality of life, cognitive function, and daily activity

Secondary outcomes including health-related QOL (QLQC30), cognitive function (MoCA), and daily activity metrics (Fitbit) are summarized in Supplemental Table 2. Health-related QOL, cognition, and daily activity remained stable at 8 and 16 weeks post-baseline with no statistically significant differences observed. Some QLQC30 items, including appetite loss, constipation, and nausea/vomiting, worsened over time, but differences were not statistically significant. Increases in quality-of-life data (QLQ-C30) and decreases in subscales for severity of seizures (BN20) (mean difference: -5.0, *p* = 0.5) and headaches (mean difference − 4.7, *p* = 0.5), were observed, but did not reach statistical significance. MoCA scores also increased over the course of the study in 13 of 17 patients with a mean difference of + 0.7 at 16 weeks from baseline, but were not statistically significant (*p* = 0.6). At 16 weeks from baseline, average daily steps taken (+ 514 steps) and night-time sleep duration (+ 0.8 hours) increased from baseline, although this change did not reach statistical significance. (Supplemental Table 2.)

### Imaging

**Fig. 5:**
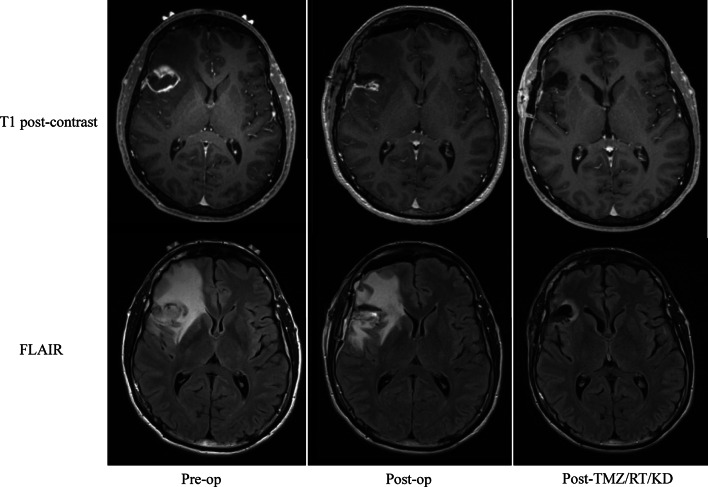
Representative MRI scan before surgery, after surgery, and after RT/chemotherapy/KD initiation. Decreased contrast enhancement and T2/FLAIR hyperintensity is noted on the post-treatment MRI.

Given all patients received concomitant standard-of-care therapy, it is not possible to readily discern the effects of KD on conventional MRI from the effects of recent and/or concomitant radiation therapy and chemotherapy. For GBM patients in particular, increased contrast enhancement within the first 3 months following the completion of concurrent radiation and temozolomide treatment is frequently observed as an effect of treatment (so-called “pseudoprogression”). Contrast enhancement also often incompletely characterizes the full extent of tumor involvement, as nonenhancing T2/FLAIR hyperintense signal adjacent to contrast enhancement often portends tumor involvement as well. We did anecdotally observe that the degree of T2/FLAIR hyperintensity on the post-chemoradiation MRI scan, which typically represents a combination of cerebral edema, radiation effect, and non-enhancing infiltrative tumor, appeared to be diminished compared to what is typically observed clinically. (Fig. 5).

## Discussion

Though the idea is not new, increasing scientific evidence supports the hypothesis that KD may be therapeutically beneficial for cancer patients, perhaps particularly so for patients with brain tumors. Many cancers, including GBM, primarily utilize glycolysis to meet the biosynthetic, bioenergetic, and signaling requirements for oncogenesis. Limiting glycolytic flux by following KD, therefore, may have anti-cancer effects, including abrogation of the insulin-PI3K signaling pathway that is aberrantly activated in most GBMs^20,21^, and facilitation of an anti-tumor immune response^22–24^. Ketones themselves may play a therapeutic role by altering oxidative stress^25^, blocking the NLRP3 inflammasome^26,27^, and acting as an endogenous inhibitor of histone deacetylase^28^. A multi-omics analysis of mouse livers revealed that multiple metabolic pathways involved in tumorigenesis, including glycolysis and the tricarboxylic acid cycle, were altered by lysine β-hydroxybutyrylation, a type of post-translational modification induced by KD^29^. Recent research has also revealed that glioma cells interact with neighboring neurons, forming glutamatergic synapses that promote glioma growth and invasiveness^30^. KD has been shown to reduce neuronal hyperexcitability and reduce glutamate signaling^31^. Collectively, the effects of KD may be protective for normal cells and exacerbate stress in tumor cells^30^. Far from being “just a diet,” KD has the potential to be a powerful lever with far-reaching consequences on both systemic and tumor physiology, as well as overall well-being^32–38^.

When it comes to diet as a treatment for cancer, historically there has been a disconnect between the enthusiasm of patients and the scarcity of clinical data, which, for brain tumor patients, is limited to a handful of small, prospective studies^8,39–43^. This has led patients to try any number of dietary and nutritional interventions -- including unsupervised quasi-KDs -- on their own, with little added to our collective body of knowledge. To minimize the gap between knowledge and hope, prospective clinical trials are needed. Regardless of whether these studies show benefit or lack thereof, they will influence clinical practice and patient decision-making.

To date, a handful of small clinical trials utilizing KD for cancer patients has been performed. Schmidt et al. conducted a pilot study looking at KD on advanced cancer patients and QOL^44^. Attrition rate was high, but patients who were able to maintain the diet reported improved emotional functioning and less insomnia^44^. Cohen et al. conducted a small, randomized trial of KD vs. American Cancer Society (ACS) diet for women with ovarian or endometrial cancer. Patients on KD had improved physical component summary scores and more energy compared to patients on the ACS diet^35^. In a study by Khodabakhsi et al., patients with local or advanced breast cancer on chemotherapy were randomized to a KD or control diet for 12 weeks^13^. Those on KD demonstrated better global QOL, improved physical activity at 6 weeks, and maintained role functioning and social functioning scores versus control^45^. A study of 25 patients with grade 2–4 astrocytoma who were treated with an 8-week diet that combined intermittent fasting with a modified Atkins diet demonstrated reductions in serum insulin levels and increased cerebral ketone levels as measured by magnetic resonance spectroscopy^46^.

KD is well tolerated by most individuals, even those on cancer directed treatment^47–49^. Known short-term side effects include weight loss, hypoglycemia, constipation, diarrhea, gastrointestinal upset, and “keto flu”-like symptoms^50^. These symptoms are more common at diet initiation and taper off shortly after. A meta-analysis of 2795 patients on KD reported kidney stones in 7.9% of adult patients^51^. The long-term effects of KD are less clear, though the lifelong use of KD for patients diagnosed with epilepsy during childhood suggests a reasonably positive long-term safety profile. Some studies report positive effects on lipids (especially HDL) and blood pressure, while other studies report increases in total cholesterol, LDL, and triglycerides with use of KD^48,52–55^.

The primary objective of this single-institution single-arm phase 1 trial was to assess the safety of implementing KD in patients with newly diagnosed GBM receiving standard chemoradiation, with a major secondary objective of feasibility. With 17 patients evaluable for analysis at the time of this publication, both objectives have been met. KD was well-tolerated, and AEs potentially attributable to the diet were generally mild. Feasibility was defined in our protocol as the ability of *≥* 50% of evaluable patients to maintain ketosis for *≥* 50% of the 16-week intervention. All 17 evaluable patients met this benchmark, with 14 maintaining ketosis > 85% of days on study. It is worth noting that the high level of adherence in this trial stands somewhat in contrast to data reported in a recent small trial conducted in the United Kingdom of KD for GBM patients, in which adherence was a challenge^8^. Reasons for this difference are unclear, although it would be interesting to investigate whether differences in implementation, patient support, and cultural influences may have played a role. Regardless, the fact that KD was safe, feasible, and well-tolerated in this trial fits with our broader clinical experience with brain tumor patients and KD, as described in our case series^9^. It is worth mentioning that our patient population was extremely motivated as seen by how quickly patients were able to achieve and maintain ketosis, as well as the privilege of having access to a plethora of grocery stores and diet related products living in the Los Angeles area.

In our study, blood glucose and ketone levels were assessed twice daily, uploaded to a data portal, and reviewed regularly by the study dietitian. By contrast, earlier oncology trials evaluating KD were more limited in their ability to incorporate real-time monitoring, with ketosis being assessed with less-accurate urine strip testing^56^. The degree of monitoring and oversight integrated into this trial allowed the study dietitian to tailor diet composition to the needs of the patients, which we believe had a positive impact on improving adherence, tolerability, and possibly outcomes. We were also able to limit patient burden by not requiring patients to keep detailed food diaries, as we had the ability to objectively monitor dietary adherence by measuring ketosis directly.

While patients were able to maintain ketosis throughout the study and glucose levels at end of study were lower than baseline, the greatest effect of KD on these parameters was observed within the first 8 weeks of the study. This may reflect common challenges adhering to KD for extended periods of time. However, it is noteworthy that 10 of 17 patients opted to continue some version of KD following completion of the study, suggesting that longer term adherence is still potentially feasible. One unanticipated consequence of conducting a diet trial in a motivated patient population was that many patients began transitioning towards a KD even prior to signing informed consent or receiving education from the study dietitian. Although this lead-time gap was small (usually just days), it is reflected in the daily ketone monitoring data, as some patients were already in ketosis at the time of their “baseline” assessment. Observed increases in morning ketone levels were statistically significant at 8 weeks, but not 16 weeks. Exploratory analysis in this small patient cohort did not find any significant association between PFS/OS and glucose levels, ketone levels, or GKI. It has been theorized that the optimal GKI for therapeutic benefit is < 2,^57^ but this benchmark has not been validated in a clinical setting. In this patient cohort, average daily GKI < 2 was achieved in 8.9% of measurements. Average GKI and ability to maintain ketosis was affected by steroid use, which was present in almost half of the cohort (47%). Steroid use impacts blood glucose and insulin levels, making it more difficult to achieve ketosis. We observed statistically significant differences in average GKI values and percent time in ketosis when comparing those receiving any steroid or higher steroid doses to those who were not on steroids. Achieving higher levels of ketosis would likely require either a stricter version of KD (e.g., 4:1), avoiding glucocorticoids, or additional pharmacotherapy or supplements (e.g., exogenous ketones), all of which would likely impact tolerability and feasibility.

Both PFS and OS in this trial compare favorably with historical data for GBM. The landmark EORTC/NCIC phase 3 trial that established temozolomide as part of standard initial treatment demonstrated median PFS of 6.9 months and OS of 14.6 months from time of diagnosis^58^. The median PFS and OS observed in this phase 1 trial were 12.9 months and 29.4 months respectively, calculated from time of diet initiation, which occurred a median of 56 days after diagnosis. While encouraging, given the small number of patients, whether a KD truly impacts long-term outcomes requires further study.

Most GBM patients who implement KD are primarily motivated by a desire to prolong survival, an outcome for which KD is not yet proven to achieve. However, even in the absence of a survival benefit, for this patient population, a cost-effective intervention that can help maintain or improve QOL would be also impactful and clinically relevant. The incidence of adverse effects in this study is comparable to adverse effects typically seen with standard radiation and temozolomide treatment. Although QOL and cognitive test changes over time were not statistically significant, they also remained stable and improved in some cases, which is contrary to the decline in QOL, cognition, and physical function commonly experienced by this population during treatment^1,59^. QOL scores were also higher than the reference ranges for a general brain cancer population^60,61^. These findings, in conjunction with the data demonstrating good tolerability and adherence, should assuage concerns that KD represents an excessive burden in this patient population.

This small, single-arm, phase 1 trial has several limitations. Although the median PFS and OS observed in this trial are impressive, the trial was not powered to evaluate efficacy. To encourage inclusivity, patients were allowed to enroll in the trial from the time of diagnosis up until the initiation of post-radiation chemotherapy, which potentially selects for a trial cohort that is more motivated and trial-seeking, with higher performance status compared to the newly diagnosed GBM patient population at-large. It is worth noting, however, that baseline patient characteristics match up well with the landmark phase 3 EORTC/NCIC trial that defined the existing SOC. (Supplemental Table 3.) Patients also received standard treatment under the direction of their treating oncologist; thus, the relative contribution of KD to survival outcomes is yet undetermined. Although patients were closely monitored by a dietitian for this study, precise macronutrient analysis of diet intake was not performed. We also note that DEXA scans were not performed to quantify effects on body composition. Additionally, in this small study, there was no statistically significant relationship between survival and blood glucose, ketone levels, or GKI.

Although study patients tolerated KD well, implementing and monitoring KD is a time- and effort-intense endeavor for patients, caregivers, and study staff alike. The increasing availability of keto-friendly meals and snacks, as well as an expanding array of keto-friendly recipes, have helped make KD a more feasible and palatable option than the inpatient regimens used previously. For many, though, the diet is still a significant departure from dietary habits engrained over decades. It is also worth noting that all the patients enrolled in this trial were White with only one patient of Hispanic ethnicity. Future trials in this space should make concerted efforts to enroll patients of diverse backgrounds and overcome any barriers to participation.

While the results reported are encouraging, much remains unknown: If KD is indeed beneficial, what is the optimal target level for ketosis? Is the benefit derived from lowering circulating glucose, the presence of ketones, or both? Do exogenous ketones provide benefit? What tumor- and patient-related factors affect outcome and approach to treatment? What treatments (e.g., immunotherapeutic, metabolic, or targeted therapies) have the potential to synergize with KD? Our experience suggests that clinical responses to KD are not uniform, and it is possible that some patients benefit while others do not. What distinguishes responders from non-responders, and how can this knowledge be used for therapeutic advantage? These questions and others need to be addressed with well-conducted translational studies and prospective randomized clinical trials. Building on these findings and outstanding questions, we have launched an NCI-funded multicenter randomized controlled phase 2 trial of KD versus standard dietary guidance along with standard-of-care chemoradiation for patients with newly diagnosed GBM that is powered to evaluate efficacy (NCT05708352).

## Electronic supplementary material

Below is the link to the electronic supplementary material.

Supplementary Material 1

Supplementary Material 2

## Data Availability

The data that support the findings of this study are available from the corresponding author, J.H., upon reasonable request.
