# Supplementary material for: A phase 1 safety and feasibility trial of a ketogenic diet plus standard of care for patients with recently diagnosed glioblastoma
Source: Sci Rep. 2025 Jul 1;15:21064. doi: 10.1038/s41598-025-06675-6 (PMC12215994; doi:10.1038/s41598-025-06675-6)
Supplement: Supplementary file 1 — Supplementary Material 1 [file 41598_2025_6675_MOESM1_ESM.docx]

**Supplemental Figure 1**. Study Schema


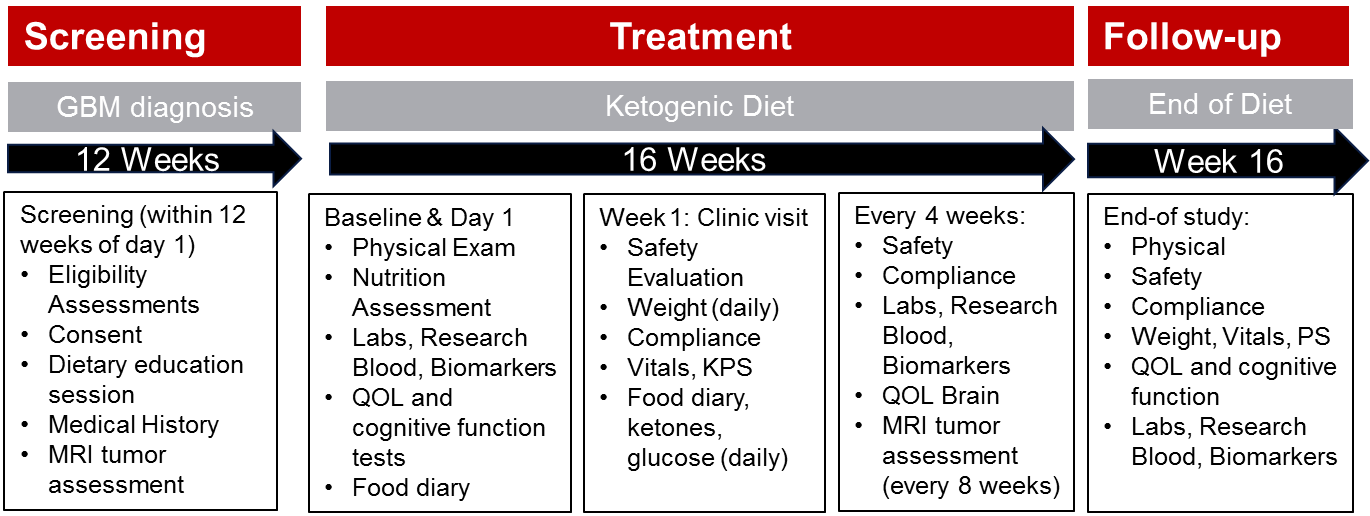


**Supplemental Figure 2**. Changes in ketones, glucose, and BMI over time using a penalized smoothing spline

Estimated change with 95% confidence intervals in A) Ketones B) Glucose C) BMI over itme

1. Ketones


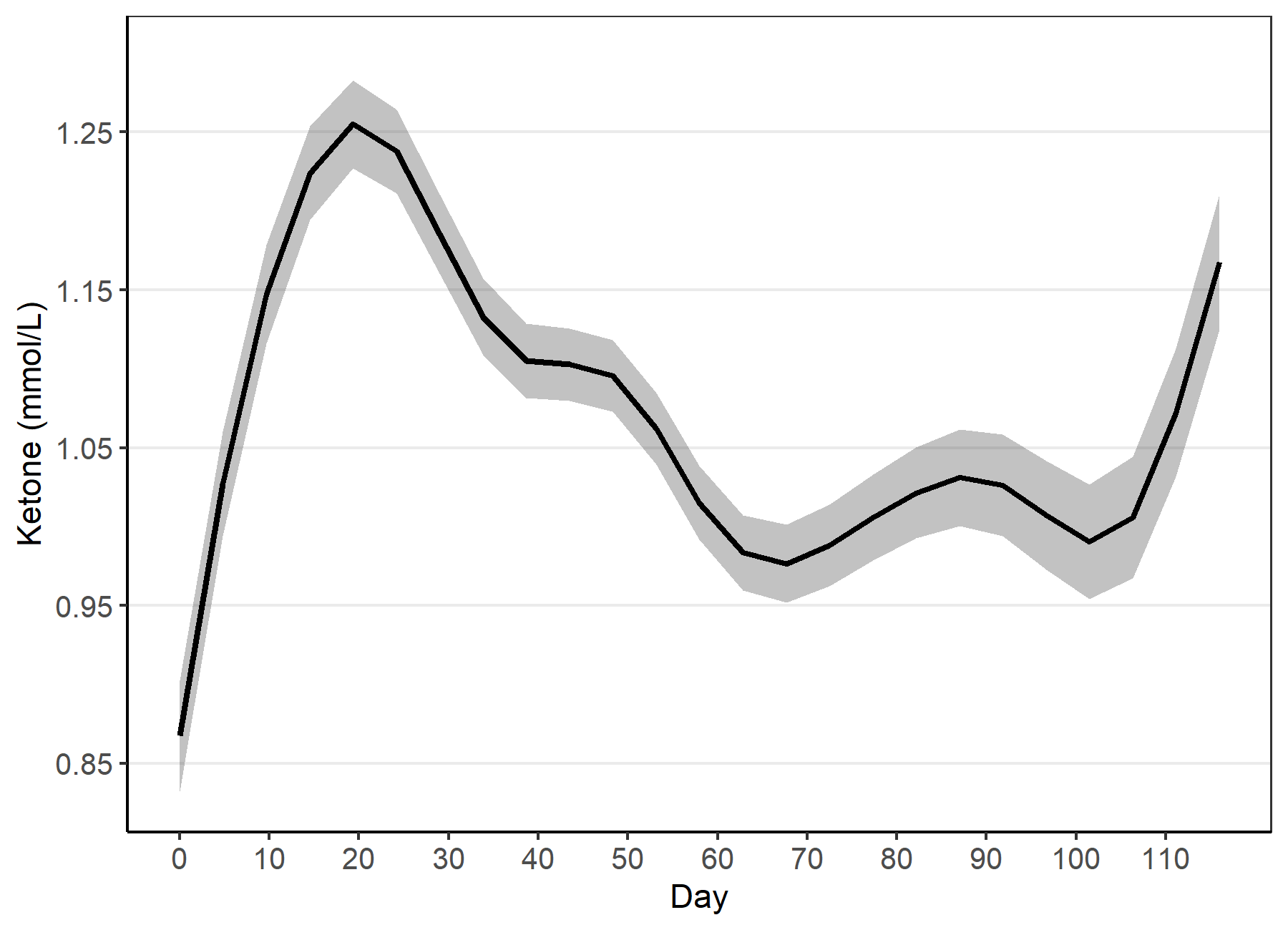


1. Glucose


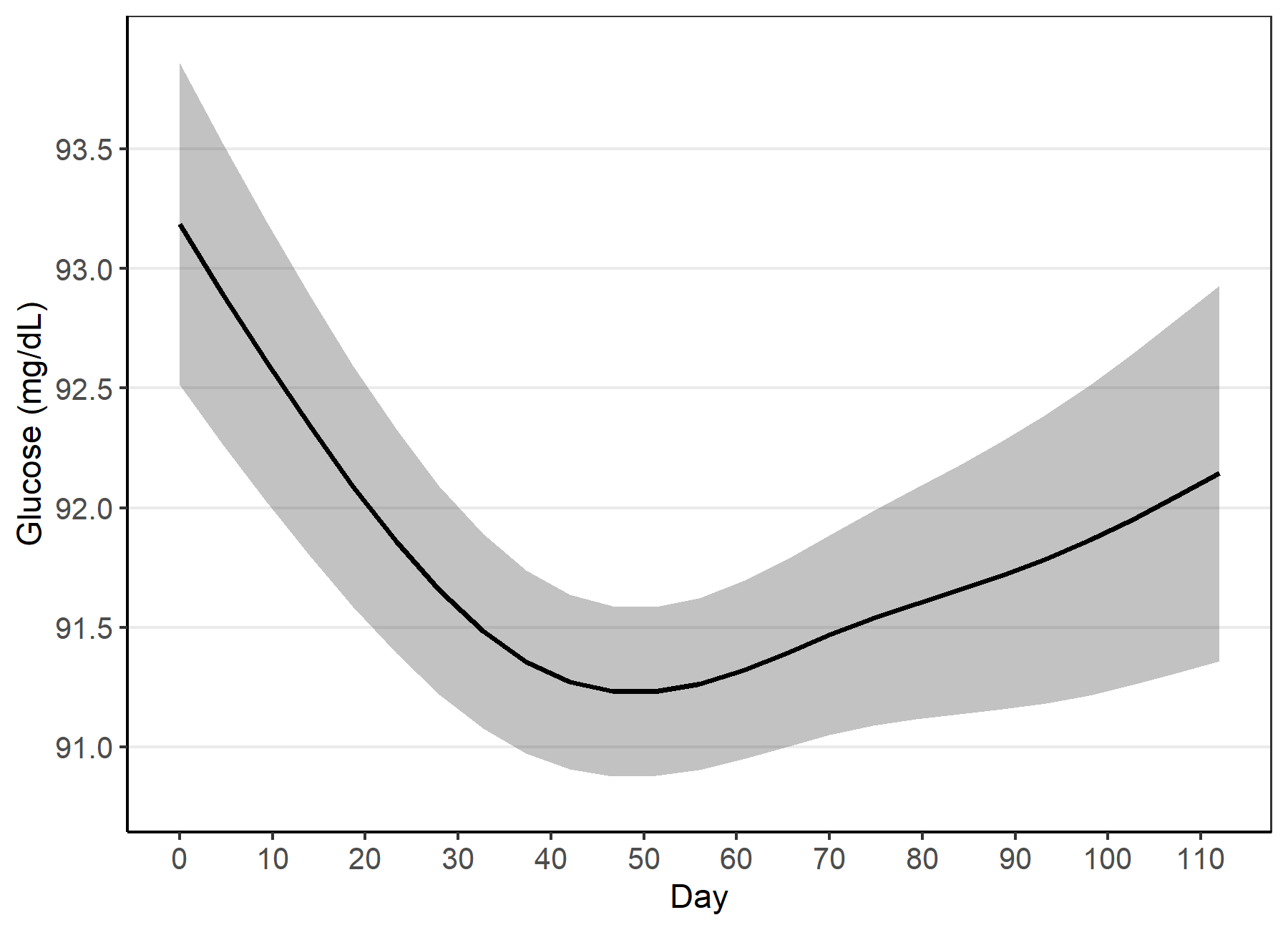


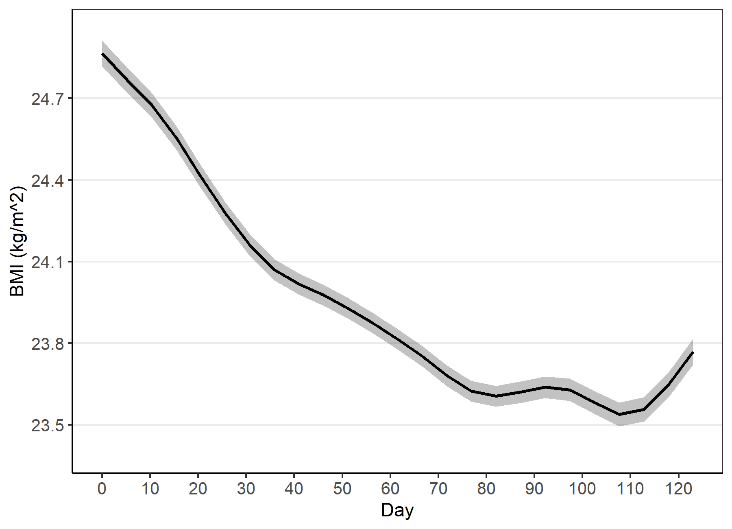
C) BMI

*Bolded line indicates estimated mean values with 95% confidence intervals (shaded area)

**Supplemental Figure 3.** Effect of ketogenic diet on inflammatory markers (IGF-1 and Insulin)
