# Supplementary material for: A phase 1 safety and feasibility trial of a ketogenic diet plus standard of care for patients with recently diagnosed glioblastoma
Source: Sci Rep. 2025 Jul 1;15:21064. doi: 10.1038/s41598-025-06675-6 (PMC12215994; doi:10.1038/s41598-025-06675-6)
Supplement: Supplementary file 2 — Supplementary Material 2 [file 41598_2025_6675_MOESM2_ESM.docx]

**Supplemental Table 1.** Univariable analysis for Progression Free Survival (PFS) and Overall Survival (OS)

|  | | **PFS** | | | **OS** | | |
| --- | --- | --- | --- | --- | --- | --- | --- |
| **Variable** | | **HR** | **CI** | **p-value** | **HR** | **CI** | **p-value** |
| **Age (Years)** | |  |  |  |  |  |  |
|  | Continuous | 1.08 | 0.99-1.18 | 0.092 | 1.12 | 1.0-1.26 | 0.041 |
| **KPS** |  |  |  |  |  |  |  |
|  | Continuous | 1.05 | 0.97-1.15 | 0.24 | 1.01 | 0.94-1.09 | 0.76 |
|  | >90 | REF |  |  |  |  |  |
|  | <90 | 1.8 | 0.53-6.32 | 0.96 | 1.30 | 0.38-4.34 | 0.34 |
| **Gender** | |  |  |  |  |  |  |
|  | Female | REF |  |  | REF |  |  |
|  | Male | 1.15 | 0.34-3.84 | 0.83 | 1.28 | 0.38-4.32 | 0.69 |
| **MGMT methylation** | |  |  |  |  |  |  |
|  | Methylated | REF |  |  | REF |  |  |
|  | Unmethylated | 10.6 | 1.31-85.3 | 0.03 | 2.42 | 0.61-9.5 | 0.13 |
| **Steroid Use** | | 0.72 | 0.21-2.5 | 0.6 | 2.42 | 0.64-9.12 | 0.2 |
| **Average ketone levels (continuous)** | | 0.75 | 0.33-1.69 | 0.48 | 0.72 | 0.27-1.92 | 0.5 |
| **% days ketones >.0.3** | | 0.99 | 0.96-1.03 | 0.66 | 0.99 | 0.96-1.03 | 0.66 |
| **Average glucose levels (continuous)** | | 1.09 | 0.91-1.23 | 0.22 | 1.06 | 0.92-1.25 | 0.43 |
| **Average GKI (continuous)** | | 0.99 | 0.93-1.06 | 0.80 | 1.01 | 0.98-1.04 | 0.38 |
|  | |  |  |  |  |  |  |

**Supplemental Table 2**. Mean change in patient-reported outcomes, cognitive function, and activity

|  | **Baseline (N=17)** | | **Week 8**  **(N=17)** | | **Week 16 (End of Study) (N=17)** | | **Mean differences** | | | |
| --- | --- | --- | --- | --- | --- | --- | --- | --- | --- | --- |
| **Variables** | **Mean (SD)** | | **Mean (SD)** | | **Mean (SD)** | | **Change**  **W8-BL** | **p-value** | **Change**  **W16-BL** | **p-value** |
| **QLQ-C30^2^** |  |  |  |  |  |  |  |  |  |  |
| Physical function | 88.6 (14.9) | | 88.2 (16.1) | | 84.0 (15.5) | | -0.4 | 0.9 | -4.6 | 0.5 |
| Role function | 70 (35.7) | | 74.5 (25.8) | | 71.8 (19.7) | | 4.5 | 0.68 | 1.8 | 0.8 |
| Cognitive function | 77.5 (23.5) | | 76.5 (22.1) | | 74.4 (24.2) | | -1.0 | 0.9 | -3.1 | 0.7 |
| Emotional function | 84.3 (15.8) | | 81.7 (15.8) | | 81.4 (21.0) | | -2.6 | 0.6 | -2.9 | 0.7 |
| Social function | 73.5 (22.9) | | 67.6 (29.1) | | 78.2 (25.8) | | -5.9 | 0.5 | 4.7 | 0.6 |
| **Sub-scales** |  |  |  |  |  |  |  |  |  |  |
| Fatigue | 35.3 (26.1) | | 37.9 (24.6) | | 35.9 (24.5) | | 2.6 | 0.8 | 0.6 | 0.9 |
| Pain | 14.7 (19.4) | | 6.9 (11.9) | | 17.9 (20.9) | | -7.8 | 0.2 | 3.2 | 0.7 |
| Appetite loss | 9.8 (25.7) | | 23.5 (32.8) | | 23.1 (34.4) | | 13.7 | 0.2 | 13.3 | 0.2 |
| Constipation | 86.3 (20.6) | | 88.2 (16.4) | | 92.3 (19.9) | | 1.9 | 0.8 | 6 | 0.4 |
| Nausea/vomiting | 7.8 (16.8) | | 10.8 (14.4) | | 12.8 (16.9) | | 3 | 0.6 | 5 | 0.4 |
| Insomnia | 13.7 (20.6) | | 17.6 (23.9) | | 23.1 (25.0) | | 3.9 | 0.6 | 9.4 | 0.3 |
|  |  | |  | |  | |  |  |  |  |
| **Neurologic symptom items (QLQC30-BN)** | | |  | |  | |  |  |  |  |
| Seizures | 7.1 (26.7) | | 2.1 (8.3) | | 2.4 (8.9) | | -5.0 | 0.5 | -4.7 | 0.5 |
| Headache | 15.7 (20.8) | | 13.5 (8.3) | | 11.9 (16.6) | | -2.2 | 0.7 | -3.8 | 0.6 |
| Drowsiness | 16.2 (20.2) | | 27.1 (34.9) | | 26.2 (32.5) | | 10.9 | 0.3 | 10 | 0.3 |
| Weakness | 7.8 (11.9) | | 9.4 (13.6) | | 13.1 (19.8) | | 1.6 | 0.7 | 5.3 | 0.4 |
| **MOCA** | 23.6 (4.4) | | 23.4 (4.9) | | 24.3 (7.1) | | -0.2 | 0.9 | 0.7 | 0.6 |
| **COWAT** | 32.2 (9.6) | | 30.5 (12.1) | | 32.6 (9.1) | | -1.7 | 0.7 | 0.4 | 0.9 |
| **Remotely-monitored activity (Fitbit)** | | | | | | |  |  |  |  |
| Average steps/day | 6030 (2495) | | 5749 (2789) | | 6544 (4889) | | -282 | 0.8 | 514 | 0.7 |
| MVPA (min) | 14.3 (10.1) | | 10.9 (13.3) | | 15.1 (12.4) | | -3.4 | 0.4 | 0.8 | 0.9 |
| Sedentary time (hours) | 13.5 (3.8) | | 15.2 (5.3) | | 13.6 (4.3) | | 1.7 | 0.3 | 0.1 | 0.9 |
| Sleep (hours) | 6.6 (0.93) | | 6.1 (2.6) | | 7.4 (1.5) | | -0.5 | 0.5 | 0.8 | 0.1 |
| Number of sleep disturbances | 10.0 (6.5) | | 10.6 (8.9) | | 13.0 (8.5) | | 0.6 | 0.8 | 3 | 0.6 |
| PA= physical activity; MVPA= moderate-to-vigorous physical activity | | | | | | | | | | |

**Supplemental Table 3**. Comparison of baseline characteristics, median PFS, and median OS across GBM trials.

|  | Median age | Debulking surgery (%) | Methylated MGMT promoter (%) | Median PFS (mo) | Median OS (mo) |
| --- | --- | --- | --- | --- | --- |
| Ph 3 EORTC/NCIC trial | 56 | 84% | 45% | 6.9 | 14.6 |
| ***Ph 1 Keto GBM trial*** | ***55*** | ***79%*** | ***43%*** | ***12.9*** | ***29.4*** |
